# Ankylosing spondylitis is associated with the anthrax toxin receptor 2 gene (*ANTXR2*)

**DOI:** 10.1136/annrheumdis-2014-205643

**Published:** 2014-08-28

**Authors:** T Karaderi, S M Keidel, J J Pointon, L H Appleton, M A Brown, D M Evans, B P Wordsworth

**Affiliations:** 1 National Institute for Health Research Oxford Comprehensive Biomedical Research Centre, University of Oxford, Oxford, UK; 2 National Institute for Health Research Oxford Musculoskeletal Biomedical Research Unit, University of Oxford, Oxford, UK; 3 University of Queensland Diamantina Institute, Translational Research Institute, Brisbane, Queensland, Australia; 4 MRC Integrative Epidemiology Unit, University of Bristol, Bristol, UK

**Keywords:** Ankylosing Spondylitis, Inflammation, Gene Polymorphism, Epidemiology, Spondyloarthritis

## Abstract

**Objectives:**

*ANTXR2* variants have been associated with ankylosing spondylitis (AS) in two previous genome-wide association studies (GWAS) (p∼9×10^−8^). However, a genome-wide significant association (p<5×10^−8^) was not observed. We conducted a more comprehensive analysis of *ANTXR2* in an independent UK sample to confirm and refine this association.

**Methods:**

A replication study was carried out with 2978 cases and 8365 controls. Then, these were combined with non-overlapping samples from the two previous GWAS in a meta-analysis. Human leukocyte antigen (HLA)-B27 stratification was also performed to test for *ANTXR2*-HLA-B27 interaction.

**Results:**

Out of nine single nucleotide polymorphisms (SNP) in the study, five SNPs were nominally associated (p<0.05) with AS in the replication dataset. In the meta-analysis, eight SNPs showed evidence of association, the strongest being with rs12504282 (OR=0.88, p=6.7×10^−9^). Seven of these SNPs showed evidence for association in the HLA-B27-positive subgroup, but none was associated with HLA-B27-negative AS. However, no statistically significant interaction was detected between HLA-B27 and *ANTXR2* variants.

**Conclusions:**

*ANTXR2* variants are clearly associated with AS. The top SNPs from two previous GWAS (rs4333130 and rs4389526) and this study (rs12504282) are in strong linkage disequilibrium (r^2^≥0.76). All are located near a putative regulatory region. Further studies are required to clarify the role played by these *ANTXR2* variants in AS.

## Introduction

The genetic association between ankylosing spondylitis (AS) and human leukocyte antigen (HLA)-B27 was first established 40 years ago. Subsequently, it has become clear that AS is a polygenic disease with over 40 variants at 28 loci involved.^1^ These include anthrax toxin receptor 2 (*ANTXR2*), also known as capillary morphogenesis gene 2 (*CMG2*) to take account of its functions in basement membrane matrix assembly, angiogenesis and embryonic development.^2^ Rare *ANTXR2* mutations cause the recessive Mendelian conditions juvenile hyaline fibromatosis and infantile systemic hyalinosis,^3^ ^4^ while common upstream variants show suggestive association with myopia.^5^ *ANTXR2* has no obvious role in AS, but it contains two single nucleotide polymorphisms (SNP), rs4333130 and rs4389526, which are in strong linkage disequilibrium (LD) in Europeans,^6^ ^7^ that have shown suggestive association with AS in two previous genome-wide association studies (GWAS). In 2010, the Triple A Australo-Anglo-American Spondyloarthritis Consortium (TASC) reported association with the intronic SNP, rs4333130 (p=9.3×10^−8^)^7^; and in 2011, the Wellcome Trust Case Control Consortium 2 (WTCCC2) showed an association with rs4389526 (meta-analysis p=9.4×10^−8^).^6^ In neither study did the evidence for association reach the threshold for genome-wide significance (p<5×10^−8^). Subsequently, two smaller Chinese studies failed to show association with *ANTXR2.*^8^ ^9^ Unfortunately, neither of the two strongly associated *ANTXR2* SNPs was included in the recent Immunochip study designed to replicate and refine suspected genetic associations with AS.^1^ Consequently, we sought to clarify the potential association through a more comprehensive analysis of *ANTXR2* in another independent UK sample prior to starting more detailed analysis of this region.

## Materials and methods

### Patients, controls and statistics

The study was approved by the National Research Ethics Service, Cambridgeshire 4 Research Ethics Committee, UK (MREC project number 98/5/23). We studied 2978 AS cases fulfilling the 1984 modified New York Criteria^10^ who were not in the previous TASC 2010^7^ or WTCCC2 analyses.^6^ Cases were genotyped on nine SNPs within *ANTXR2* that had previously shown evidence of association with AS (p<0.05) in the TASC 2010 study.^7^ SNPs were genotyped using KASP technology (competitive allele-specific polymerase chain reaction amplification) by LGC Genomics (Hoddesdon, UK). Genotyping assays were validated on KASP panels, and cluster plots were checked for clearly separated genotype clusters.

Cases were compared to 8365 children from the Avon Longitudinal Study of Parents And Children who had previously been genotyped on the Illumina 550K platform (rs4234848, rs11098965, rs4444771, rs10000471, rs4333130, rs6839672) and then imputed to HapMap 2 as previously described (rs12504282, rs6534639 and rs4640621; imputation quality R^2^≥0.98).^11^ ^12^ Please note that the study website contains details of all the data that is available through a fully searchable data dictionary (http://www.bris.ac.uk/alspac/researchers/data-access/data-dictionary/).

Control allele frequencies were checked for Hardy–Weinberg equilibrium (all p>0.05). Allele frequencies between cases and controls were compared by logistic regression analysis. Where SNPs were imputed, ‘best guess’ genotypes were used in the calculation (all R^2^≥0.98). All statistical analyses were carried out using using the software programme PLINK (http://pngu.mgh.harvard.edu/purcell/plink).^13^ Expected power of the study was based on the ORs from the TASC 2010 GWAS discovery sample.^7^ We estimate that our study had 98% statistical power to detect an association corresponding to an allelic OR of 0.82, with a minor allele frequency of 0.31, an α of 0.05 (two-sided), and a disease prevalence of 4/1000, assuming an additive model for disease risk (http://www.dartmouth.edu/~eugened/power-samplesize.php).

### Stratified analyses

Cases and controls were stratified by HLA-B27 status, and tested for association using logistic regression analysis. HLA-B27 status of cases and controls was determined using the rs4349859 marker, which tags *HLA-B27* in Europeans with very high sensitivity and specificity.^6^

### Meta-analysis

ORs from the TASC 2010 (1236 cases, 3979 controls), WTCCC2 2011 (1787 cases, 5162 controls) and this study were combined in an inverse variance meta-analysis assuming a fixed effects model. Combinability of the studies was determined using Cochran's Q test (p>0.05), and the inconsistency measure (I^2^) was used to estimate the amount of heterogeneity across the studies in the meta-analysis. For TASC, the analysis included correction for four principal components due to the different ethnicities in that study.^7^ Genome-wide significance level (p<5×10^−8^) was used for declaring statistically significant genetic association. Meta-analysis conditioning on rs12504282 was also performed to detect secondary signals at the locus. Forest plots were produced using the metafor package in R.

### *ANTXR2*-HLA-B27 interaction analysis

Logistic regression analyses were performed in each cohort using the model (logOdds=B_0_+HLA-B27(dominant)+*ANTXR2*(additive)+*ANTXR2*xHLA-B27). The logORs and SEs for the interaction terms were combined in an inverse variance meta-analysis to test for the presence of an *ANTXR2*-HLA-B27 interaction.

### Bioinformatics

LocusZoom was used to display a regional association plot showing the pairwise LD between the top SNP and the other SNPs in the meta-analysis (1000 Genomes March 2012 European panel).^14^ The University of California, Santa Cruz (UCSC) genome browser (http://genome-euro.ucsc.edu/index.html) was used to interrogate the Encyclopaedia of DNA elements (ENCODE) data for regions of interest containing the associated SNPs.^15^ ^16^ Public eQTL databases were used to determine possible effects of SNPs in the *ANTXR2* region on gene expression (eQTL databases: http://www.sph.umich.edu/csg/liang/imputation/17 and http://www.hsph.harvard.edu/liming-liang/software/eqtl/; GTEx: http://www.gtexportal.org; Genevar^18^: http://www.sanger.ac.uk/resources/software/genevar/).

## Results

### Replication study

Five SNPs showed nominal evidence of association (p<0.05) with AS in the expected direction (table 1A). Four of these SNPs were also nominally associated with AS in the HLA-B27-positive subgroup analysis (table 1B), whereas only one of these showed nominal evidence of association in HLA-B27-negative AS (table 1C). There appeared to be a difference in the point estimates of the ORs between HLA-B27-positive and negative individuals (with HLA-B27-positive individuals showing stronger association for most SNPs) although the CIs around the estimates were wide.

**Table 1:** Associations of 9 *ANTXR2* SNPs with AS in (1A) the independent replication study, (1B) HLA-B27-positive and (1C) HLA-B27-negative stratified analyses

Replication study											
(A) All cases vs controls (2978 vs 8365)						(B) B27+ cases vs B27+ controls (1935 vs 696)			(C) B27– cases vs B27– controls (358 vs 7669)		
SNP	Minor allele	MAF (%) cases/controls	p Value	OR	95% CI	p Value	OR	95% CI	p Value	OR	95% CI
rs6534639	C	45/47	0.0005*	0.90	0.85 to 0.95	0.09	0.90	0.79 to 1.02	0.01*	0.83	0.71 to 0.96
rs4234848	G	31/32	0.08	0.94	0.88 to 1.01	0.37	0.94	0.82 to 1.08	0.1	0.88	0.74 to 1.04
rs11098965	C	24/25	0.2	0.96	0.89 to 1.03	0.19	0.91	0.79 to 1.05	0.4	0.93	0.78 to 1.11
rs4444771	G	24/25	0.2	0.95	0.89 to 1.02	0.14	0.90	0.78 to 1.03	0.6	0.96	0.80 to 1.14
rs12504282	C	43/46	0.0001*	0.89	0.84 to 0.94	0.006*	0.84	0.74 to 0.95	0.4	0.93	0.80 to 1.08
rs10000471	C	33/35	0.01*	0.92	0.86 to 0.98	0.03*	0.87	0.76 to 0.99	0.1	0.88	0.75 to 1.04
rs4640621	A	34/36	0.001*	0.90	0.85 to 0.96	0.01*	0.85	0.75 to 0.97	1.0	1.00	0.85 to 1.17
rs4333130	G	34/36	0.002*	0.91	0.85 to 0.97	0.02*	0.86	0.75 to 0.98	1.0	1.00	0.85 to 1.17
rs6839672	A	16/15	0.6	1.02	0.94 to 1.11	0.10	1.16	0.97 to 1.38	0.3	0.90	0.72 to 1.12

Nominally significant (p<0.05) associations are shown (*).

Number of cases and controls in each analysis is also shown in brackets. (B27: HLA-B27).

HLA, human leukocyte antigen; MAF, minor allele frequency; SNP, single nucleotide polymorphism.

### Meta-analysis

**Figure 1:**
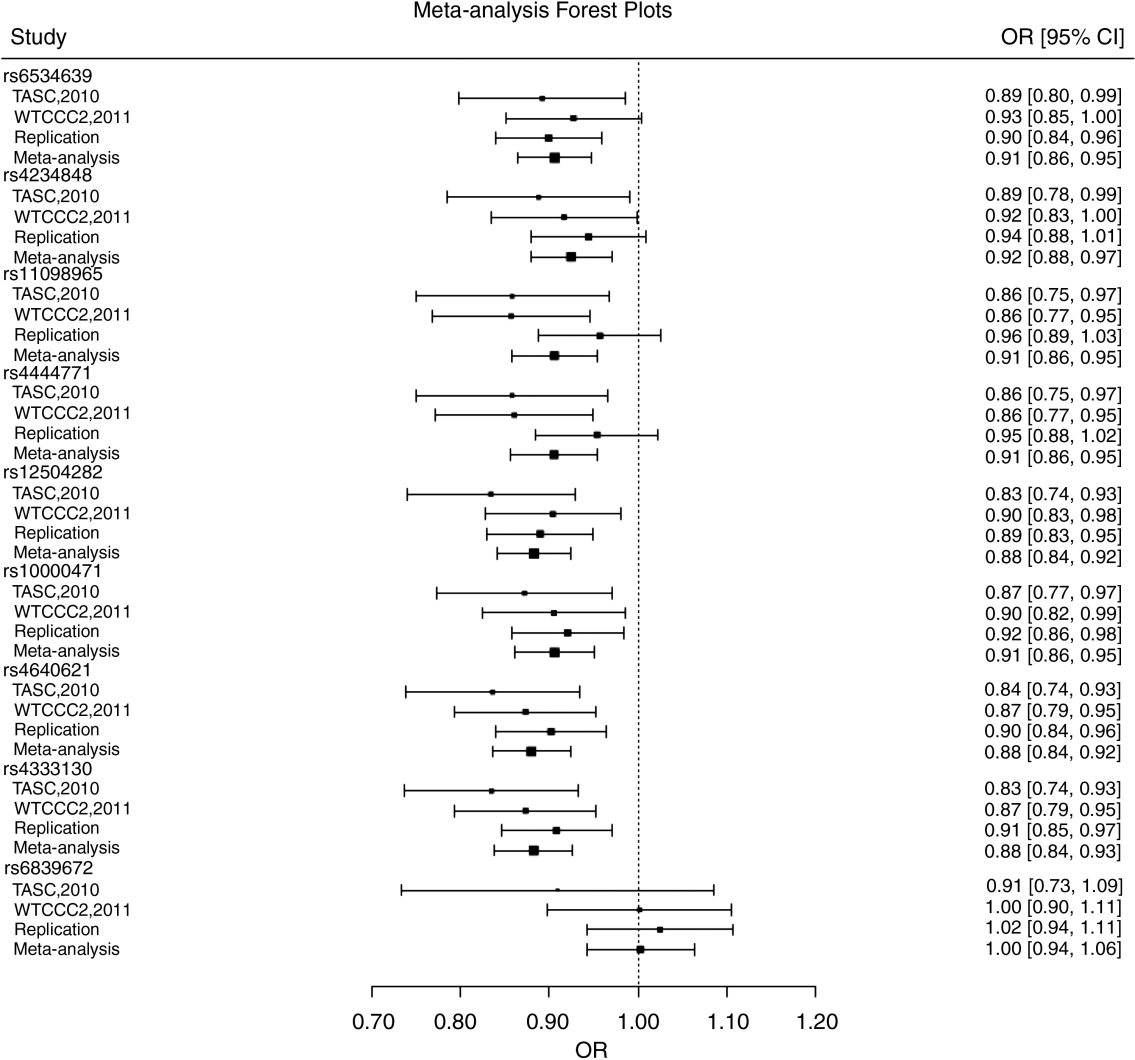
Forest plots for overall meta-analysis of 9 single nucleotide polymorphisms (SNPs). Plots for SNPs with highly significant p-values, rs12504282 (p=6.7×10^−9^), rs4640621 (p=1.2×10^−8^), rs4333130 (p=2.5×10^−8^) and rs6534639 (p=4.5×10^−6^) show agreement across the studies with overlapping CIs. Two SNPs rs11098965 (I^2^=59%, Cochran's Q p=0.09) and rs4444471 (I^2^=54%, Cochran's Q p=0.11) exhibit some degree of heterogeneity, but this was not statistically significant (p>0.05).

In the meta-analysis, three SNPs showed strong evidence of association with AS (p<5×10^−8^), while five SNPs exhibited suggestive associations (4.5×10^−6^≤p≤0.0007; table 2A). Forest plots revealed that the direction and strength of the association was consistent across most SNPs (figure 1). Conditional analysis indicated no associations independent of rs12504282 (all p>0.1). Seven of the SNPs showed nominal evidence for association in the HLA-B27-positive AS subgroup (table 2B), but none was associated with HLA-B27-negative AS (p>0.05; table 2C). However, no statistically significant interaction was detected between HLA-B27 and *ANTXR2* variants (p>0.05).

**Table 2:** Associations of *ANTXR2* SNPs with AS in (2A) the overall meta-analysis, (2B) HLA-B27-positive and (2C) HLA-B27-negative stratified meta-analyses

Meta-analysis												
(A) All cases vs controls (6001 vs 17 506)					(B) B27+ cases vs B27+ controls (4555 vs 1427)				(C) B27–cases vs B27–controls (761 vs 16 079)			
SNP	p Value	OR	Cochran's Q p Value	I^2^ (%)	p Value	OR	Cochran's Q p Value	I^2^ (%)	p Value	OR	Cochran's Q p Value	I^2^ (%)
rs6534639	4.5×10^−6^	0.91	0.77	0	0.08	0.93	0.52	0	0.13	0.92	0.08	60
rs4234848	0.0007	0.92	0.59	0	0.01	0.89	0.44	0	0.57	0.97	0.24	30
rs11098965	7.1×10^−5^	0.91	0.09	59	0.006	0.87	0.65	0	0.22	0.93	0.70	0
rs4444771	6.4×10^−5^	0.91	0.11	54	0.005	0.87	0.74	0	0.35	0.94	0.75	0
rs12504282	6.7×10^−9^	0.88	0.40	0	0.0003	0.85	0.74	0	0.56	0.97	0.68	0
rs10000471	1.2×10^−5^	0.91	0.66	0	0.0004	0.85	0.81	0	0.40	0.95	0.47	0
rs4640621	1.2×10^−8^	0.88	0.43	0	0.002	0.87	0.91	0	0.44	0.96	0.36	3
rs4333130	2.5×10^−8^	0.88	0.35	5	0.003	0.88	0.95	0	0.44	0.96	0.36	2
rs6839672	0.9	1.00	0.48	0	0.1	1.10	0.63	0	0.17	0.90	0.96	0

Three SNPs reached genome-wide significance (p<5×10^−8^) in the overall meta-analysis.

Number of cases and controls in each analysis is also shown in brackets. (B27: HLA-B27).

AS, ankylosing spondylitis; HLA, human leukocyte antigen; SNP, single nucleotide polymorphism.

## Discussion

**Figure 2:**
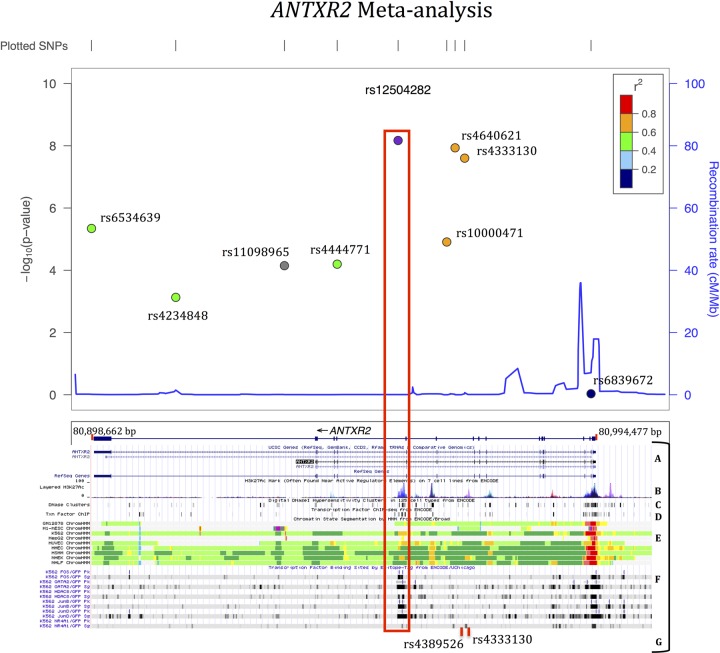
Regional association plot showing *ANTXR2* (*CMG2*) and all the single nucleotide polymorphisms (SNPs) in the study on the x-axis and their corresponding −log_10_p values on the y-axis. Historic sex-averaged recombination rates are also shown in blue (top panel). Linkage disequilibrium (r^2^, 1000 Genomes March 2012 European reference panel) between the top SNP rs12504282 (purple) and the other SNPs are also shown with colour-coding. ENCODE data showing that rs12504282 and the previous top genome-wide association studies (GWAS) SNPs are near a region of possible enhancer binding are presented in the bottom panel (A) Gene structure; (B) H3K27Ac mark, ie, often found near active regulatory elements; (C) DNaseI hypersensitivity clusters; (D) Transcription factor ChIP-seq; (E) Chromatin state segmentation; (F) Transcription factor binding sites by epitope-tag; G- Top SNPs from the previous GWAS (rs4333130 (TASC, 2010) and rs4389526 (WTCCC2, 2011)). These three top SNPs from three different studies are strongly correlated (rs4389526-rs4333130 r^2^=1.0, D′=1.0; rs4389526-rs12504282 r^2^=0.8, D′=1.0; rs4333130-rs12504282 r^2^=0.76, D′=1.0).

This study convincingly replicated the association between *ANTXR2* variants and AS in an independent UK sample with three variants reaching genome-wide significance level in the meta-analysis. In the replication study, the strongest association was with rs12504282 (p=0.0001, OR=0.89, 95% CI 0.84 to 0.94), but there is a high degree of LD across this locus and the five SNPs showing significant association (table 1A) are distributed across the locus (figure 2). In the meta-analysis, eight SNPs showed evidence of association, three SNPs reaching genome-wide significance; rs12504282 again showed the strongest association (p=6.7×10^−9^, OR=0.88). Forest plots show that direction and magnitude of association across the studies in the meta-analysis are in agreement with overlapping CIs (figure 1).

The strength of evidence for association appeared to be stronger in HLA-B27-positive patients compared with the HLA-B27 negatives. None of the seven SNPs, which showed nominal evidence of association with HLA-B27-positive AS showed nominal association with HLA-B27-negative disease, including the top SNP rs12504282 (table 1B, C). However, failure to detect nominal association with *ANTXR2* in HLA-B27-negative AS probably reflects reduced statistical power rather than a genuine epistatic interaction. Consistent with this interpretation, the *ANTXR2*-HLA-B27 interaction analysis statistically testing for an epistatic effect revealed no significant findings. By contrast, the well-established epistatic genetic effect between HLA-B27 and *ERAP1* in AS^6^ can be explained by the functional synergy between ERAP1 and HLA class I molecules in antigen processing and presentation.^19^ It is much less easy to explain a putative genetic interaction with *ANTXR2* where there is no known functional interplay with HLA-B27 at the molecular level.

Biological explanations for the consistent associations of *ANTXR2* variants with AS have not yet been forthcoming. The top SNPs from three different studies,^6^ ^7^ including this one, are in close proximity and strongly correlated (rs4333130, rs4389526 and rs12504282; r^2^>0.76). Furthermore, these SNPs are near a putative transcription factor-binding region, and may therefore affect the expression level of *ANTXR2* or another gene (figure 2). *ANTXR2* is widely expressed in many tissues. Interrogation of expressed quantitative trait loci (eQTL) databases revealed SNP rs4690110 with a strong cis-eQTL effect on *ANTXR2* expression in adipose tissue (p=7.6×10^−9^, β=0.14),^18^ but rs4690110 is weakly correlated with the top SNP rs12504282 (r^2^=0.27), and adipose tissue is not thought to be directly relevant to AS. Therefore, it is unlikely that this cis-eQTL effect explains the observed AS association in this region. However, the specific effects of the associated *ANTXR2* SNPs may differ in tissues relevant to AS. Of interest, rs4333130 and rs12504282 have trans-eQTL effects on the expression of *NSMAF* on chromosome 8 (allele A, β=−0.27, p=0.00025) and *GPR89A* on chromosome 1 (allele T, β=−0.25, p=0.00088), respectively, in lymphoblastoid cell lines.^17^ *NSMAF* encodes a WD-repeat protein potentially playing a role in regulating tumour necrosis factor-induced cellular responses, such as inflammation. *GPR89A* encodes G protein-coupled receptor 89A involved in Golgi apparatus acidification possibly modulating its functions.^20^ Substantially more work will be required before the nature of this tantalising genetic association with AS can be explained at a functional level.
